# Binding of PFOS to serum albumin and DNA: insight into the molecular toxicity of perfluorochemicals

**DOI:** 10.1186/1471-2199-10-16

**Published:** 2009-02-25

**Authors:** Xian Zhang, Ling Chen, Xun-Chang Fei, Yin-Sheng Ma, Hong-Wen Gao

**Affiliations:** 1 State Key Laboratory of Pollution Control and Resource Reuse, College of Environmental Science and Engineering, Tongji University, Shanghai 200092, PR China; 2 Key Laboratory of Yangtze River Water Environment of Ministry of Education, College of Environmental Science and Engineering, Tongji University, Shanghai, PR China 200092; 3 Environmental Engineering College, NanJing Forestry University, Nanjing 210037, PR China

## Abstract

**Background:**

Health risk from exposure of perfluorochemicals (PFCs) to wildlife and human has been a subject of great interest for understanding their molecular mechanism of toxicity. Although much work has been done, the toxigenicity of PFCs remains largely unknown. In this work, the non-covalent interactions between perfluorooctane sulfonate (PFOS) and serum albumin (SA) and DNA were investigated under normal physiological conditions, aiming to elucidate the toxigenicity of PFCs.

**Results:**

In equilibrium dialysis assay, the bindings of PFOS to SA correspond to the Langmuir isothermal model with two-step sequence model. The saturation binding number of PFOS was 45 per molecule of SA and 1 per three base-pairs of DNA, respectively. ITC results showed that all the interactions were spontaneous driven by entropy change. Static quenching of the fluorescence of SA was observed when interacting with PFOS, indicating PFOS bound Trp residue of SA. CD spectra of SA and DNA changed obviously in the presence of PFOS. At normal physiological conditions, 1.2 mmol/l PFOS reduces the binding ratio of Vitamin B_2 _to SA by more than 30%.

**Conclusion:**

The ion bond, van der Waals force and hydrophobic interaction contributed to PFOS binding to peptide chain of SA and to the groove bases of DNA duplex. The non-covalent interactions of PFOS with SA and DNA alter their secondary conformations, with the physiological function of SA to transport Vitamin B_2 _being inhibited consequently. This work provides a useful experimental method for further studying the toxigenicity of PFCs.

## Background

Biomolecules such as proteins and DNA have been a subject of great interest for the last hundreds of years because of their key functional roles in the cellular processes. Serum albumin is the major protein component of blood plasma. It is responsible for the maintenance of oncotic pressure of blood plasma [1] as well as that of blood pH [2]. It is the well-known model protein and is called a multifunctional plasma carrier protein for its ability to bind a wide variety of ligands. These include inorganic cations, organic anions, amino acids, and, perhaps most important, physiologically available insoluble endogenous compounds, e.g., fatty acids [3-7], bilirubin [1], and bile acids. The locations of the fatty acid binding sites throughout the protein have been mapped by structural study [6,7]. In fact, not only endogenous ligands but also exogenous compounds bind to HSA, for example, commonly used drugs with acidic or electronegative features, e.g., warfarin [8], camptothecins [9] and inorganic polymers such as polyoxometalates [10]. Recently, the binding mechanism of organic contaminants or toxins to HSA has been investigated, e.g. arazine [11], ochratoxin [12], methyl parathion [13], and arsenic [14]. The interactions of organic contaminants with serum albumin often cause conformational change in the protein or even the subsequent change of its physiological function. Concerning to DNA, whose conformation and sequence preference is critical to replication, transcription and DNA chromatin compaction [15], it is reported that DNA is subjected to the effect of organic chemicals, too, whether it is endogenous ligands or exogenous compounds. As the target of many drugs [16,17] and also most likely of a large number of environmental pollutants [18,19], DNA has been extensively studied about the binding mechanism such as minor groove binding, major groove binding, and (bis)intercalation. For example, Distamycin A, a well-known polyamide antibiotic, can bind in the minor groove of duplex DNA primarily at AT-rich sequences as a monomer or as a side-by-side antiparallel dimer. Its binding affinity derives from specific hydrogen bonding contacts between the amide protons and the O2 or N3 of pyrimidines and purines, respectively, electrostatic interactions with the backbone, and van der Waals contacts with the walls of the minor groove [20]. In fact, the involvement of any substances is likely to affect the activity of the biomolecule, either enhancing it [21] with potential medical significance or inhibiting it [22] if it is associated with an organic contaminant or toxin. After all, there is still a lot unknown about the binding mechanism of biomolecules.

For over half a century, perfluorochemicals (PFCs) have been extensively used in a variety of consumer and industrial applications due to their physical and chemical properties: chemical stability, thermal inertness, low surface energy and an amphiphilic nature [23,24]. Given their physical and chemical properties, PFCs have been found not only persistent in the environment, bioaccumulative through food chain but also ubiquitously distributed all over the world [25-35] including the Arctic, Antarctic and Pacific region. Several studies have been conducted on the presence and levels of PFCs in marine mammals, fish, birds, and humans [25-35]. PFOS were found in high concentrations in liver tissue in top predators, polar bears, and even in the cord serum of newborns in many countries [33,36-38]. Recently, PFCs have been identified as new persistent organic pollutants [39]. Laboratory studies have reported the developmental and reproductive toxicity [40-45], neurotoxicity [46-49], immunotoxicity [50,51] and carcinogenicity [52,53] of PFOS in rodents both *in vivo *and *in vitro*. At cellular, molecular and gene levels, PFOS was found to cause peroxisomal proliferation [54,55], change of membrane surface potential [56], mitochondrial dysfunction [57], disturbance of fatty acid metabolism [58], hepatocellular hypertrophy [59] and even change of gene expression [60]. In 2000, 3 M, the major manufacturer of these compounds, announced the discontinuation of its perfluorooctane-based compounds due to concerns regarding persistence, worldwide dissemination, toxicity, and bioaccumulation.

Elucidation of the mechanism of biomolecular interactions such as protein-protein [61] and DNA-ligand binding [16,17], enzyme catalysis, and inhibition is crucial to understanding of cellular processes including signal transduction, gene regulation, and enzyme reactions [62]. Conventional molecular spectrometric methods such as fluorescent probe, UV, and circular dichroism (CD) have been widely used for the investigation of protein-ligand interaction. Recently, binding mechanisms have been studied using equilibrium dialysis, x-ray crystallography, NMR, isothermal titration calorimetry (ITC), and surface plasmon resonance biosensors [63,6,17,65], which are powerful analytical tools in enzymology, rational drug design, and toxicology. In this study, equilibrium dialysis, fluorophotometry, ITC and CD were used to characterize the non-specific interactions between PFOS and SA/DNA in the normal physiological condition, pH 7.4 and 0.15 M electrolyte and 37°C. The object is to analyze the interaction forces, sites and type and then further understand the toxigenicity of PFCs.

## Results and discussion

### Characterization of the interactions of PFOS with SA and DNA

HPLC-MS was used to validate the simple colorimetric detection method for the determination of PFOS during dialysis process. The two methods have achieved the consistent results in the measurement of three PFOS dialysis solutions (see Additional File 1). Therefore, the colorimetric method is feasible for the determination of PFOS in the present work. The interaction of PFOS (L) with SA and DNA (M) can be summarized below:

(1) $$\begin{array}{llllll} & N\text{L} & + & \text{M} & \to & {\text{ML}}_{\text{N}}\\ \text{Initiation} & c_{\text{L0}} & & c_{\text{M0}} & & \text{0}\\ \text{Equilibrium} & c_{\text{L}}=c_{\text{L0}}-Nc_{\text{M0}} & & c_{\text{M}}\to \text{0} & & c_{\text{M0}} \end{array}$$

Both *c*_L0 _and *c*_M0 _are the initial concentrations of PFOS and of SA or DNA pipeted into dialysis bags. The symbol *c*_L _is the equilibrium concentration of PFOS, *N *the saturation binding number of PFOS, *K*_*b *_the binding constant. Considering PFOS was diluted 4 times during the equilibrium dialysis, the effective fraction (*f*) and the molar ratio (γ) of PFOS bound to SA or DNA can be calculated by the relationships below [66]:

(2) $$f=1-\frac{4\times c_{L}}{c_{L0}}$$

and

(3) $$\gamma =\frac{f\times c_{L0}}{c_{M0}}$$

It can be deduced that

(4) $$\gamma =\frac{c_{L0}-4c_{L}}{c_{M0}}$$

**Figure 1:**
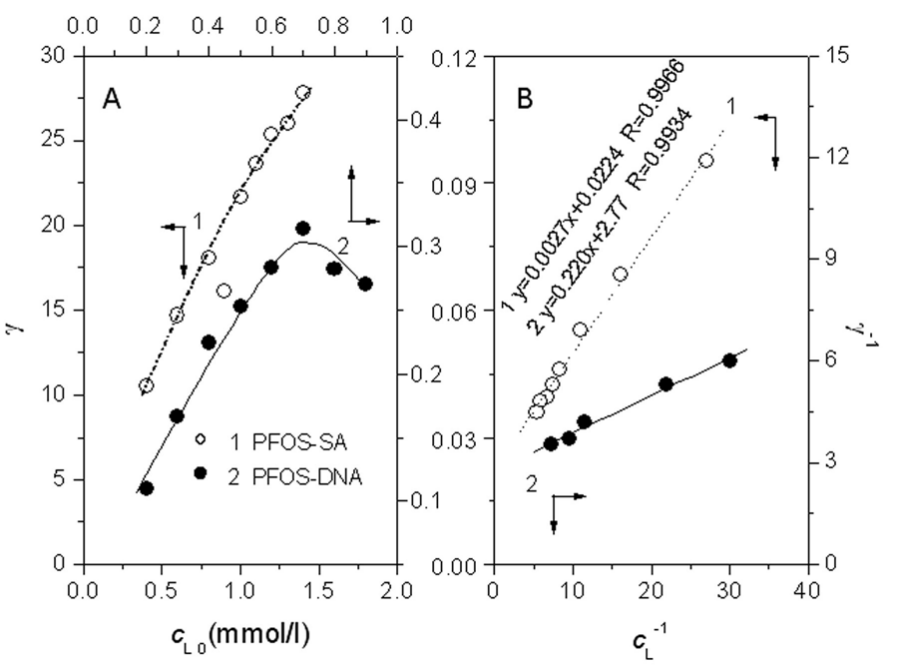
**Plots of (A) γ vs. *c*_L0 _and (B) γ^-1 ^vs. *c*_L_^-1 ^for the PFOS solutions containing 0.02 mmol/l SA (1) and 0.96 mmol/l DNA (2) at pH7.40 at 37°C in the presence of 0.15 mol/l NaCl**.

By measuring a series of PFOS solutions containing known concentrations of SA or DNA, γ was calculated using Eq. 4. As is shown in Fig. 1A, the γ value increases with increasing PFOS concentration. The Langmuir isothermal model below was used to fit the experimental data.

(5) $$\frac{1}{\gamma }=\frac{1}{N}+\frac{1}{KNc_{L}}$$

where *K *is the adsorption constant. The plots of γ^-1 ^versus *c*_L_^-1 ^are shown in Fig. 1B. The good linear relationships of the plots indicate that the bindings of PFOS to peptide chains and DNA duplex obey the Langmuir isothermal model. The interactions of PFOS with SA and DNA are the chemical adsorption in monolayer. From the intercepts of the regression lines in Fig. 1B, *N *of PFOS molecules bound per molecule of SA and per bp of DNA was calculated to be 45 and 0.36, respectively.

The electrostatic attraction plays an important role in the binding of sulfonic ligands to proteins. Several studies in our laboratory have revealed that electrostatic attraction between the sulfonic groups of azo compounds and the positively charged amino acid residues (AARs) of SA is the main contributor that fixes the position of these compounds in protein in acidic media and induces the subsequent combined actions of multiple non-covalent bonds, e.g., hydrogen bonds, hydrophobic interactions and van der Waals force [67,68]. Given the dissociation constants (*K*_R_) of the side groups (R) of basic and acidic AARs of SA (10.53 for Lys, 6.00 for His, 12.48 for Arg, 3.65 for Asp, and 4.25 for Glu), only the side groups of Lys and Arg residues are protonated and positively charged in neutral solutions in our study, so selectrostatic attraction between the sulfonic acid group of PFOS and positively charged AARs of SA will be much weaker than that in acidic media. But concerning the high polarity of PFOS, the electrostatic attraction between the sulfonic acid group of PFOS and the positively charged -NH_3_^+ ^groups of Lys and Arg of SA can still play an important role in the PFOS-SA binding [69,70]. Moreover, the hydrophobic interaction will also occur between the long alkyl group of PFOS and the nonpolar side groups of AARs of SA such as Lys, Arg, His and so on. Similarly, PFOS may bind to DNA via hydrophobic interaction resulting from the long alkyl group and the homolateral bases in the groove of DNA.

### Effects of pH, electrolytes and temperature

**Figure 2:**
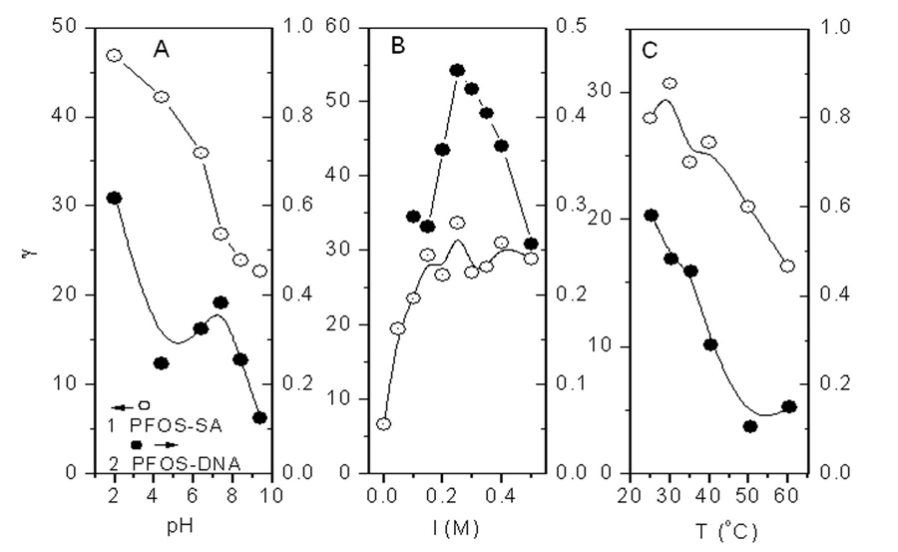
**Effects of pH (A), electrolyte (B) and temperature (C) on γ of solutions containing 0.8 mmol/l PFOS, 0.016 mmol/l SA (A and B) and 0.02 mmol/l SA (C), 0.9 mmol/l DNA (A) and 1.19 mmol/l DNA (B and C)**.

The stability of non-covalent interaction is always affected by various environmental conditions such as pH, ionic strength and temperature [71,72]. As is shown in Fig. 2A, γ for the PFOS-SA binding decreases obviously with increasing pH. It implies that the acidic media are more favorable for PFOS binding to SA. As we know from the dissociation constants (*K*_R_) of the side groups (R) of basic and acidic AARs of SA, more side groups (R) of AARs are protonated and positively charged in acidic media. Thus, the electrostatic attraction between the sulfonic acid head of PFOS and the positively charged AARs will be much stronger than that in neutral media [67,68], which result in the increasing of binding number of PFOS to SA. Moreover, SA tends to unfold in acidic media, so steric hindrance decreases, leading to an increasing of possible binding sites as well as the binding number. As to DNA, increasing binding is obviously observed at pH 2. Similar to SA, DNA tends to unfold in acidic media, too. The increasing of possible binding sites resulting from the unfolding of DNA may be the reason that PFOS-DNA binding number increases. In addition, a peak of γ appears at pH 7.4 for the PFOS-DNA binding and then γ decreases at pH 8.4 and 9.4 afterwards. It implies the neutral media is comparatively more favorable for PFOS binding, indicating the potential risk of PFOS toxicity under the physiological condition of wildlife and human.

Sodium chloride was added in the PFOS-SA/DNA solutions to investigate the effect of electrolyte on the non-covalent interaction. Human blood normally contains approximately 0.15 mol/l electrolyte. Although γ of PFOS-SA binding (Fig. 2B) increases when the electrolyte is less than 0.15 mol/l, γ is only slightly different in 0.15 mmol/l electrolyte from that in 0.25 mol/l electrolyte. This implies that ions in human blood would not affect PFOS binding. From the curves in Fig. 2B, a peak of γ of the PFOS-DNA binding appears at 0.25 mol/l electrolyte, indicating that ions in body fluids will affect the PFOS-DNA binding more or less.

High temperature has two opposing effects on non-covalent interaction. On one hand, the peptide chain and DNA duplex will expand with heating and the three-dimensional conformation will be favorable for the insertion of organic molecules. On the other hand, such expansion will increase the distance between peptide chains and DNA duplex, leading to the redistribution of the effective binding sites on the peptide chain and DNA duplex and the subsequent desorption of small organic substances [73]. The balance between these mechanisms decides the effect of temperature on the non-covalent interactions. As is shown in Fig. 2C, γ values for the PFOS-SA/DNA interactions decrease with increasing of temperature. Obviously the distance between the peptide chains or DNA duplex increases as a result of temperature increase and lead to the desorption of PFOS, which result from the subsequent decrease of binding sites for PFOS. Therefore, γ values of the PFOS-SA/DNA interactions decrease.

### The intrinsic fluorescence analysis of Trp residues of SA

The intrinsic fluorescence analysis of tryptophan residue (Trp) of SA is often used in the investigation of a ligand-protein binding. The quenching of protein fluorescence depends on the degree of exposure of Trp residue to the polar, aqueous solvent and its proximity to specific quenching groups such as protonated carboxyl, protonated imidazole and deprotonated ε-amino groups [74]. Two models have been proposed for the quenching of protein fluorescence: static quenching and dynamic quenching [75]. In static quenching, the quencher binds to Phe, Tyr, especially Trp residues of SA and consequently the compound in ground state that has no fluorescence is formed, resulting in the decrease in the fluorescence intensity of SA. On the contrary, in dynamic quenching the decrease in the fluorescence intensity of SA is only because of the molecular collision between the quencher and SA. Higher temperatures usually result in faster diffusion, collision and hence larger amounts of dynamic quenching. In order to confirm the quenching mechanism, the fluorescence quenching data presented here was assumed to be dynamic quenching. The classical relationship often employed to describe the dynamic quenching process is the Stern-Volmer equation:

(6) $$\frac{F_{0}}{F}=1+K_{q}\tau_{0}[Q]$$

**Figure 3:**
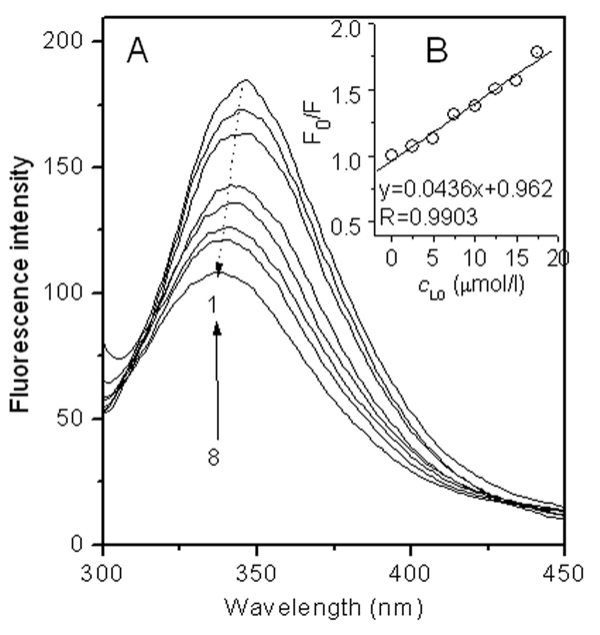
**(A) The fluorescence spectra of solutions containing 0.5 μmol/l SA and variable PFOS from 1 to 8: 0, 2.5, 5, 7.5, 10, 12.5, 15, 17.5 μmol/l at pH7.40**. (B) Plot of F_0_/F vs. Q for the SA-PFOS solutions.

Where F_0 _is the fluorescence intensity of SA without PFOS, F is the fluorescence intensity of SA in the presence of PFOS, τ_0 _is the lifetime of the fluorophore of SA in the absence of quencher, usually 10^-8 ^s for biomolecule, and *Q *is the concentration of PFOS (mol/l), the quencher, *K*_q _is the bimolecular quenching rate constant and 2.0 × 10^10 ^l/(mol·s) is the highest value of *K*_q _for dynamic quenching. In this study if *K*_q, PFOS-SA _(*K*_q _for PFOS-SA binding) is calculated to be much larger than 2.0 × 10^10 ^l/(mol·s), static quenching occurs for PFOS-SA binding; if *K*_q, PFOS-SA _is calculated to be less than 2.0 × 10^10 ^l/(mol·s), dynamic quenching occurs. By regression of plots *F*_0_*/F *vs *Q *(Fig. 3B), the relationship *y *= 0.0436*x *+ 0.962 (R = 0.9903) was arrived. The *K*_*q*_τ_0 _value could be equivalent to 0.0436 (PFOS in umol/l in Fig. 3B). *K*_q, PFOS-SA _is calculated to be 4.36 × 10^12 ^l/(mol·s), which is much greater than the highest dynamic quenching constant, 2.0 × 10^10^l/(mol·s). So the interaction between PFOS and SA resulted in static quenching. Moreover, the fluorescence spectra of SA were obtained in the presence of PFOS (Fig. 3A). Not only a gradual decrease in the fluorescence intensity of SA but also a blue shift (10 nm) in the emission wavelength were observed, indicating that PFOS bound to the Trp residue, W214 of SA and the microenvironment around Trp, Phe and Tyr residues was changed consequently. This phenomenon confirmed the occurrence of static quenching between PFOS and SA again.

SA contains a hydrophobic cavity and all hydrophobic AARs were present in the cavity [76], including Trp residue, which is favorable for hydrophobic group to enter. PFOS enters the cavity and interacts with the hydrophobic AARs via hydrophobic interaction between the alkyl group of PFOS and the aromatic group of AARs, causing the quenching of SA fluorescence. In addition, concerning the high polarity of the sulfonic acid group of PFOS, the electrostatic attraction could occur between the sulfonic acid group of PFOS and -NH_3_^+ ^of Lys and Arg residues of SA.

### Variation of the secondary conformation of SA and DNA

The combinations of covalent and non-covalent interactions among a protein or DNA result in the specific conformation and the corresponding function of biomolecules. When organic compounds such as a pollutants, drugs or toxicants interact with protein or DNA, the internal non-covalent bonds of the peptide chain or DNA duplex are often disrupted, possibly changing the original conformation or even their special function. CD spectrometry is often used to evaluate the secondary structure of DNA [77] or a protein [68], such as the fractions of β-pleated sheet, α-helix and β-turn.

**Figure 4:**
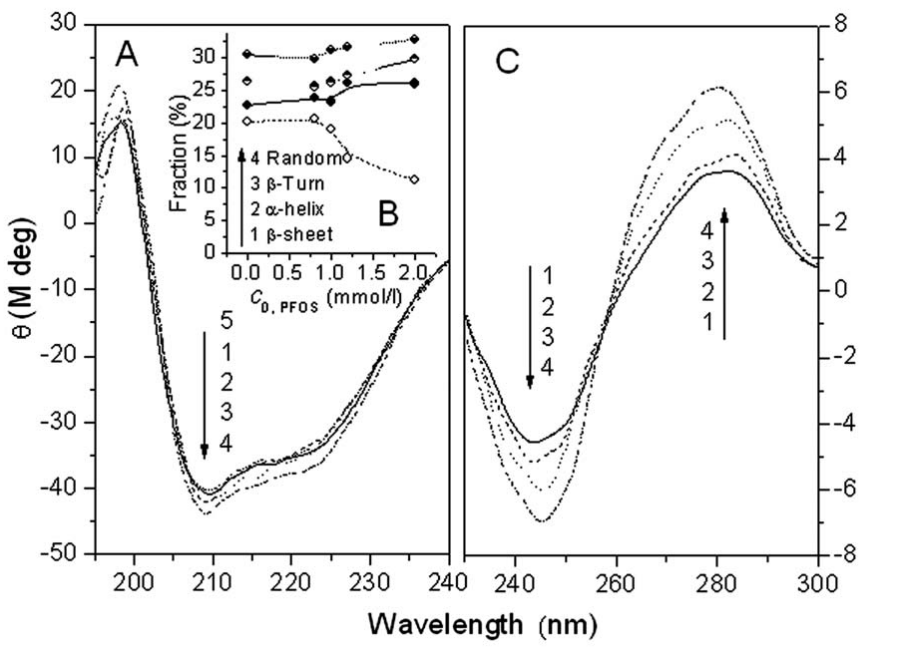
**The molar ellipticity CD curves of solutions containing (A) 0.4 μmol/l SA in the presence of PFOS from 1 to 5: 0, 16, 20, 24, 40 μmol/l and (C) 100 μmol/l DNA with PFOS from 1 to 4: 0, 20, 30, 40 μmol/l; (B) Plot of fraction (%) vs. C_0, PFOS _for the SA-PFOS solutions**. All solutions were prepared at pH7.40.

The molar ellipticity CD curves of PFOS-SA/DNA solutions are given in Fig. 4. As is shown in Fig. 4B, the fractions of β-pleated sheet, α-helix, and β-turn of SA changed obviously in the presence of PFOS. With the addition of PFOS, the β-pleated sheet fraction of SA decreases from 20.2% to 11.3% whereas that of α-helix and β-turn increases from 22.8 to 26.1% and 26.5 to 29.8%, respectively. Obviously the disappearance of β-pleated sheet results in the increase of α-helix and β-turn content. Although in neutral solutions the non-covalent bonds between PFOS and SA are much weaker compared with that in acidic solutions [67,68], the addition of PFOS causes obvious change of secondary structure of SA. From the data above, the addition of PFOS transforms some β-pleated sheet into α-helix and β-turn.

The CD spectra of DNA in the 230–300 nm scope show a signal characteristic of a B-form helix with a negative band at 245 nm and a positive band at 275 nm. The enhancement of CD spectra at both 245 and 275 nm in the presence of PFOS and the shape change of the positive and negative band (Fig 4C) indicated that PFOS binds in the groove of DNA. On account of the hydrophobic alkyl group of PFOS, PFOS may bind in the groove of DNA paralleling with the phosphate backbone via van der Waals force and hydrophobic interaction. Thus, the electron cloud of bps becomes expanding owing to the reversed pulling of PFOS so as to the enhancement of CD of DNA at 275 nm.

### Thermodynamic characterization of the interactions of PFOS with SA and DNA

Relating thermodynamic parameters to structural and biochemical data allows a better understanding of the mechanism of the biomolecule-ligand reaction. The ITC measurements provide information on thermodynamic quantities such as enthalpy and heat capacity changes during the molecular interaction based on the heat produced by reactions In recent years, ITC measurements have been widely applied to study e.g. protein-ligand interaction [78], DNA-ligand interaction [77].

The ITC experiments were conducted by injecting PFOS solutions into the ITC cell containing SA or DNA in pH 7.40 media at 37°C. In each experiment, an exothermic heat pulse was detected following each injection. Its magnitude progressively decreased until a plateau is reached indicating saturation of binding. The heat involved at each injection was corrected for the heat of dilution, which was determined separately by injecting the PFOS solutions into the B-R buffer and then divided by the number of moles injected. Values for the equilibrium binding constant (*K*_b_), enthalpy change (Δ*H*), and entropy change (Δ*S*) of the reaction were obtained and calculated by the Gibbs free energy (Δ*G*) equation:

(7) Δ*G *= -*RT*ln *K*_*D *_= Δ*H *- *T*Δ*S*

**Figure 5:**
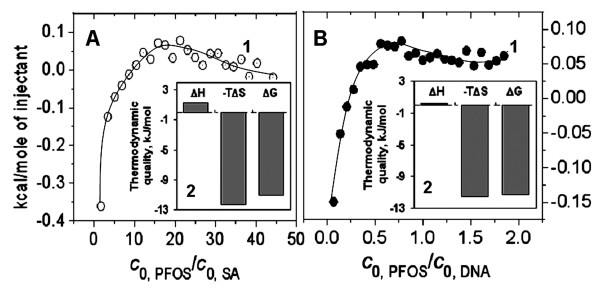
**ITC titration curves of (A) PFOS-SA and (B) PFOS-DNA interactions at pH7.40**. The temperature was 37°C. The experiment was conducted by injecting: 2.5 mmol/l PFOS (10 μl every time) into the ITC cell (1.4685 ml) containing (A) 0.01 mmol/l SA or (B) 0.5 mmol/l DNA. The titration profile was integrated and corrected for the heat of dilution, which was estimated by a separate experiment by injecting the PFOS into the B-R buffer. The corrected heat was divided by the moles of injectant, and values were plotted as a function of the PFOS/SA and PFOS/DNA molar ratio. The titration curve was fitted by a nonlinear least squares method.

As is shown in Fig. 5, all Δ*H *are much less than 60 kcal/mol, so the PFOS-SA/DNA interactions are non-covalent [79], involving the electrostatic attraction, hydrophobic interaction, and van der Waals force. Moreover, the heat released from both PFOS-SA (Fig. 5A) and PFOS-DNA (Fig. 5B) interactions approaches zero when c_L0_/c_M0 _> 10 and 0.3, respectively, indicating the plateau is being reached. All the reactions are driven by entropy change and thus spontaneous because Δ*H *is much less than that -*T*Δ*S *(Fig. 5B-2).

**Figure 6:**
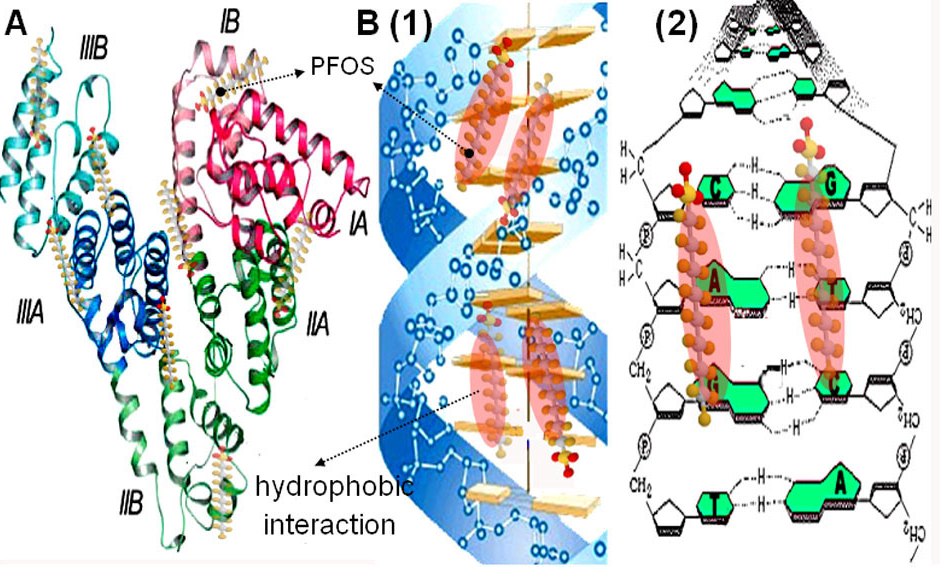
**Cartoon illustrating the possible binding sites of PFOS in SA (A) and DNA (B)**.

SA is composed of three homogenous and helical domains (Fig. 6A). Every domain consists of two sub-domains, which face each other like two notches. All the hydrophobic amino acid residues embedded in the sub-domains formed three hydrophobic cavities [2]. During protein-ligand interaction, polar bonds are likely to be formed outside of the hydrophobic cavities because polar residues of SA predominate here. The reactions are usually exothermic due to the high energy of polar bond. On the contrary, hydrophobic interactions are likely to occur inside of the hydrophobic cavity. The reactions are usually endothermic with the folding of protein and decreasing of entropy change.

From Fig. 5A, the binding of PFOS to SA corresponds to two-step sequence model: on the surface and inside the cavity. First, the heat released from PFOS-SA interactions was negative when c_L0_/c_M0 _< 10, indicating the occurrence of exothermic reaction and the formation of polar bonds. Because the polar bonds are more likely to be formed outside of the hydrophobic cavity of SA, it is suggested that PFOS binds to the surface of SA via electrostatic attraction between the sulfonic acid group of PFOS and -NH_3_^+ ^of Lys and Arg residues of SA (Fig. 6A). After the binding sites of SA surface had been occupied, PFOS molecules entered the cavity of SA easily (Fig. 6A). When c_L0_/c_M0 _> 10, the heat released from the PFOS-SA interaction was positive, indicating the occurrence of endothermic reaction and hydrophobic interaction. Because the hydrophobic interaction is more likely to occur inside of the hydrophobic cavity of SA, it is suggested that PFOS enters hydrophobic cavity of SA and binds to the nonpolar AARs there. For example, PFOS interacts with the aromatic side group of Trp residue of SA. Owing to the small cavity where inside binding occurred and the consequent effect on SA conformation, SA folds and its conformation changes.

As is shown in Fig. 5B, Δ*H *is very small. Most of the heat released from the PFOS-DNA interaction was positive, indicating the occurrence of endothermic reaction and hydrophobic interaction. PFOS probably interacts hydrophobically with the homolateral bases of DNA via the alkyl group, paralleling with the phosphate backbones of DNA (Fig. 6B). The conformation of DNA changed owing to the hydrophobic interaction that pulls the contiguous bps closer. This is confirmed from high Δ*S *values (Fig. 5B-2).

### Effects of PFOS on the physiological function of SA to transport Vitamin B_2_

The relationship between structural transformation of protein and its functioning is of great significance in organisms. During interaction process, a small organic compound may bind to the peptide chain, regulating its three-dimensional structure and even changing its corresponding function [80,81]. Although non-covalent binding is often weak and non-specific, a combination of many non-covalent bonds may alter the conformation and function of the protein [82]. Serum albumin is the most abundant protein of blood plasma. It is responsible for the maintenance of both the oncotic pressure and pH of blood. Moreover, it is the major plasma carrier protein in blood which can bind a large number of ligands, e.g., amino acids, vitamins, fatty acids, drugs and so on.

**Figure 7:**
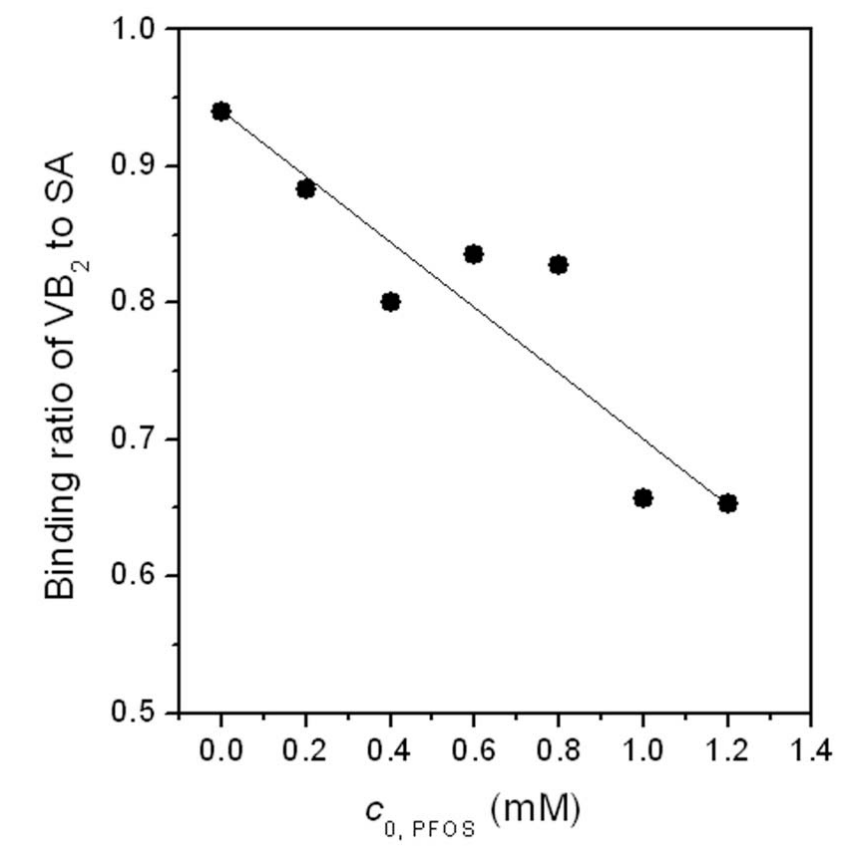
**Effect of PFOS on the physiological function of SA to transport VB_2_**. All solutions contained 0.02 mmol/l SA and 0.04 mmol/l VB_2 _at pH 7.40 at 37 °C in the presence of 0.15 mol/l NaCl.

The effects of PFOS on SA function were determined as shown in Fig. 7. With the addition of PFOS, the binding ratio of VB_2 _to SA decreases obviously comparing to that in the absence of the pollutants and it indicates the inhibition of SA carriage capacity by the pollutants. At the normal physiological condition, 1.2 mmol/l PFOS reduces the binding ratio of VB_2 _to SA by more than 30%. It's likely that the competition of binding sites in SA by pollutants and VB_2 _and the subsequent conformation change of SA are unfavorable for the binding of VB_2 _to SA. Therefore, non-covalent binding of the organic compound severely affects the physiological function of protein by altering its conformation and overlapping its active sites.

## Conclusion

By characterizing the interaction of PFOS with SA and DNA using various methods, some important results were obtained concerning e.g., binding number, binding energy, and type of binding. The interactions of PFOS with SA and DNA obeyed the Langmuir isothermal model and the saturation mole numbers of PFOS was calculated to be 45 per molecule of SA and 0.36 per bp of DNA. These numbers indicate that PFOS has a high binding capacity especially for SA in vitro. As to the toxicity of PFOS in vivo at concentrations currently observed in the environment, further research needs to be conducted. Both the interactions are driven by entropy increase and spontaneous. The electrostatic attraction and hydrophobic interaction are suggested to occur in PFOS binding to the peptide chain and the grooves of DNA. The combined action of non-covalent bonds results in change of the secondary structure of both SA and DNA, with the binding ratio of VB_2 _to SA being reduced by more than 30%, indicating the transport function of SA was inhibited consequently. This work provides a useful experimental method for studying the interaction of PFCs with biomacromolecules so as to understand the toxigenicity of PFCs.

## Methods

### Instruments and Materials

A Model F-4500 fluorospectrophotometer (Hitachi High-Technologies Cooperation, Tokyo, Japan) was used for the concentration measurement of vitamin B2 and fluorescence measurement of protein solutions in the presence of PFOS. A Model Lambda-25 spectrometer (Perkin-Elmer, Shelton, CT 06484, USA) was used to determine the concentration of PFOS during the process of equilibrium dialysis. The spectrometer was computer-controlled using UV WinLab software (Version 2.85.04). The Isothermal Titration Calorimetery (ITC) experiments were carried out on a Model VP-ITC system (MicroCal Inc., USA) with VPViewer 2000 (Version 1.04.0018). A Model J-715 CD spectropolarimeter (Jasco Instruments, Tokyo, Japan) with secondary structure estimation-standard analysis measurement software (715/No. B014460524, Jasco) was used to determine the conformation of protein and DNA. Model RC 30-5K semi-permeable membranes (Molecular Weight Cut Off 5 KDa, Shanghai Green Bird STD) were used for equilibrium dialysis. A Model DK-8D electrothermic multiporous constant temperature water-bath (Shanghai Yiheng Technol., Shanghai, China) was used in the temperature experiment. Solution pHs were measured with a Model pHS-25 acidity meter (Shanghai Precise Sci. Instrum., Shanghai, China). An Accela U-HPLC-system connected to a TSQ Quantum Access mass spectrometer (Thermo Fisher Scientific, America) was used to validate the CPC-ECR colorimetric method used for PFOS determination in this study.

Both 0.100 mmol/l SA (Sigma, A7906) and 3.7 mmol/l bps of DNA (Shanghai Chemical Reagents Company, Shanghai, China) were prepared in deionized water as a stock solution and stored at 4°C. The precise concentration of protein and DNA were determined by the UV method. PFOS stock solution (10 mmol/l) was prepared by diluting 1 ml of PFOS water solution (40% in water, Fluka, USA) in 100 ml of deionized water and stored at 4°C. Vitamin B_2 _stock solution (0.1276 mmol/l) was prepared by dissolving 12 mg of Vitamin B_2 _(VB_2_) (purity 99%, Shanghai Chemical Reagents Company, Shanghai, China) in 250 ml of deionized water and stored at 4°C. The 1.25 mol/l NaCl and Britton-Robinson (B-R) buffer (pH 7.40) containing 0.040 mol/l phosphoric acid, acetic acid and boric acid was used for the experiments.

### Determination of PFOS with CPC and ECR

PFOS reacts with cetylpyridinium chloride (CPC) by ion-pair binding to form the PFOS-CPC complex [83]. The anionic ligand, eriochrome cyanine R (ECR) was used to replace CPC from the PFOS-CPC complex to form a CPC-ECR complex. The color change was used for determination of PFOS only in this work. The replacement procedures are followed. 2.0 ml pH 3.80 acetate buffer, 0.30 ml 1.00 mM CPC and 0.50 ml 1.00 mM ECR were mixed in 5-ml calibrated flasks. After mixing for 15 min, various amounts of PFOS were added and the solutions diluted to 5 ml. After 10 min, the absorbance of each solution was measured at 626 nm against a reagent blank without PFOS.

### Equilibrium of PFOS dialysis and working curve of PFOS determination

**Figure 8:**
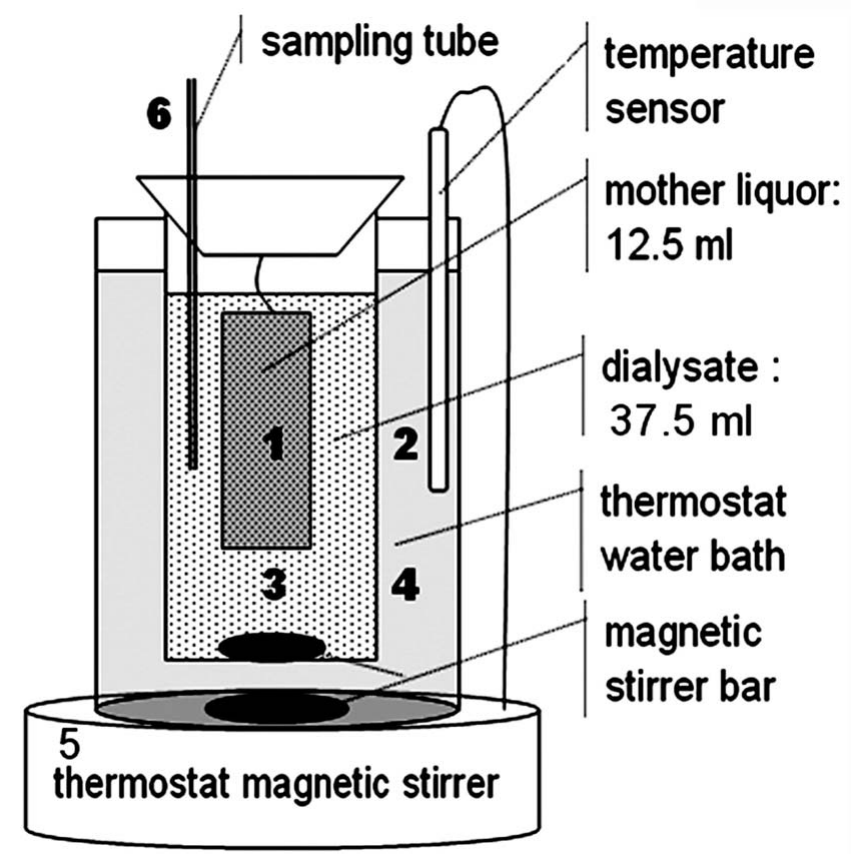
**The device designed for equilibrium dialysis**. (1) – semi-permeable membrane with 12.5 ml of dialysate; (2) – the temperature sensor for maintaining the reaction at constant 37°C; (3) – Dialysis solution 37.5 ml; (4) – water bath at constant 37°C. The apparatus was placed on a thermostated magnetic stirrer (5) and rotary magnets were used to mix solutions (3) and (4) thoroughly. The PFOS concentration in solution (3) was determined from the sampling tube (6).

**Figure 9:**
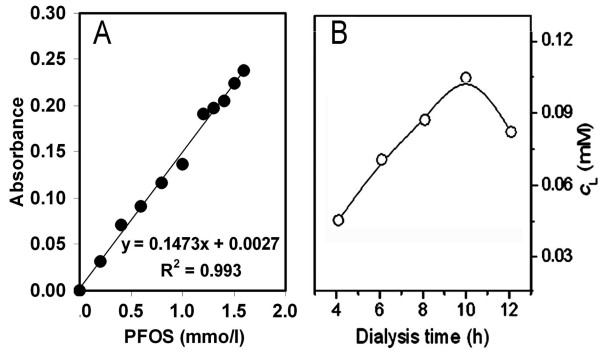
**(A) Working curve for determination of PFOS between 0 and 1.60 mmol/l according to the dialysis procedures at pH 7.40 in 0.15 mol/l NaCl and the detection method with CPC and ECR, measured at 626 nm**. (B) Effect of dialysis time on recovery of PFOS in the mother liquor containing 0.40 mmol/l PFOS at pH 7.40 in 0.15 mol/l NaCl.

A schematic diagram of equilibrium dialysis experiment was shown in Fig. 8. For equilibrium dialysis assay of PFOS, 12.5 ml mother liquor containing 2.5 ml of B-R buffer, 0.15 mol/l NaCl, a known volume of PFOS and deionized water was pipeted into dialysis bags (1). 37.5 ml of the dialysate solution containing 0.15 mol/l NaCl, 7.5 ml of B-R buffer and deionized water was added to the dialysis bag (3). The temperature of the water bath (4) was kept constant at 37°C by adjusting the thermostat magnetic stirrer (5). 1 ml of the dialysis solution (3) was collected into 5-ml calibrated flask every 2 h from the sampling tube (6). According the above detection method, the PFOS concentrations (*c*_L_) of the dialysis solutions were determined. Variation of *c*_L _with the dialysis time was shown in Fig. 9B. The dialysis of PFOS remains at equilibrium between 8 and 12 h. After dialysis for 10 hours, 1 ml of the dialysis solution (3) was collected in 5-ml calibrated flasks from every sampling tube (6). According to the detection method above, the absorbance of the dialysis solution was measured at 626 nm. The working standard curve of PFOS was established as shown in Fig. 9A. By five repetitive determinations of 0.200 mmol/l PFOS, the result was 0.199 ± 0.006 mmol/l PFOS. The recovery rates of the detection method are between 96.5 and 102% and the relative standard deviation 3%. The detection method was used for the determination of PFOS in the dialysis equilibrium solution, where no complicated interference substance co-existed.

To validate the above detection method, a high performance liquid chromatography (HPLC) system coupled to a triple quadrupole mass spectrometer (MS) was used with the method being modified [31] (Additional File 2). After equilibrium dialysis of 100.0, 150.0, 200.1 mg/l PFOS in SA presence at 37°C for 10 hours, 1.00 ml of the dialysis solution was collected and determined by the above detection method. Meanwhile, another 1.00 ml of the dialysis solution was also collected and determined by HPLC-MS after being diluted for 1000 times. Then a total of 10 μl was injected onto a Hypersil Gold C18 column (150 × 2.1 mm, 5 μm, Thermo) with a 5 mM ammonium acetate (pH 3.5)/methanol mobile phase starting at 10% methanol. At a flow rate of 250 μL/min, the gradient increased to 95% methanol at 13 min before reverting to original conditions at 16 min. Column temperature was maintained at 25°C. For quantitative determination, the HPLC system was interfaced to a TSQ Quantum Access (Thermo) mass spectrometer employing electron spray ionization in the negative ion mode and highly selective reaction monitoring. The fragment ions for PFOS m/z 499 (C_8_F_17_SO_3_^-^) were monitored for quantification.

### Equilibrium dialysis of PFOS in SA and DNA presences

In order to determine the interactions of PFOS with SA or DNA, B-R buffer, 0.15 mol/l NaCl, a known volume of SA or DNA solution (*c*_M0_), deionized water and a known volume of PFOS solution (*c*_L0_) were added in dialysis bags (1). The temperature (2) of the water bath (4) was kept constant at 37°C. Using the same method, 1 ml of the dialysis solution (3) was collected in 5-ml calibrated flasks from every sampling tube (6). According to the detection method above, the absorbance of the dialysis solution was measured at 626 nm and *c*_L _of PFOS was calculated by the working curve. γ of PFOS to SA or DNA was calculated and data were plotted.

### Fluorescence measurement of PFOS-SA solutions

The B-R buffer (2.00 ml) and 0.050 ml of 0.100 mmol/l SA were mixed with 0, 2.5, 5, 7.5, 10, 12.5, 15, 17.5 μmol/l of PFOS in 10-ml calibrated flasks. The solutions were diluted to 10 ml with deionized water and their fluorescence intensities were measured at the excitation wavelength (280 nm) and the emission wavelengths (300–450 nm).

### CD measurement of SA and DNA conformation in the presence of PFOS

The B-R Buffer (1 ml), 0.04 ml SA (0.100 mmol/l) were mixed with 0, 16, 20, 24, 40 μmol/l PFOS in flasks. The solutions were diluted to 10.0 ml with deionized water. Each sample was allowed to equilibrate for 15 min before measurement. CD spectra were taken on a spectropolarimeter with a 0.1 cm light path cell at 25°C. The mean residue ellipticity (θ) of SA was measured between 190 and 240 nm. From the θ curves, the relative contents of secondary structure forms of SA, α-helix, β-pleated sheet, β-turn and random coil, were calculated in all solutions. The 100 μmol/l DNA containing 0, 20, 30 and 40 μmol/l PFOS were measured between 190 and 240 nm using the same method.

### ITC characterization of interactions of PFOS with SA and DNA

ITC experiments were carried out as follows: the PFOS solution (2.50 mmol/l in pH 7.40 B-R buffer) was injected about 27 times in 10-μl increments at 270-S intervals into the isothermal cell containing SA (0.010 mmol/l in pH 7.40 B-R buffer) or DNA (0.500 mmol/l in pH 7.40 B-R buffer). The cell temperature was kept at 37°C. Heats of dilution of PFOS, obtained separately by injecting PFOS into the buffer, were used to correct the raw data. The corrected heats were divided by the number of moles injected and analyzed using the Origin software (version 7.0) supplied by the manufacturer. The titration curve was fitted by a nonlinear least-squares method and Δ*H *and Δ*S *were determined.

### Effect of PFOS on the physiological function of SA to transport Vitamin B_2_

The fluorescence intensity of Vitamin B_2 _(VB_2_) solution was measured at the excitation wavelength (440 nm) and the emission wavelength (525 nm) against deionized water using fluorospectrophotometer. The determination of the VB_2_-SA interaction was investigated according to the same equilibrium dialysis method as described above.

For the equilibrium dialysis assay of PFOS-VB_2_-SA, 12.5 ml solution containing 2.5 ml of B-R buffer, 0.15 mmol/l NaCl, 0.02 mmol/l SA (*c*_M0_), 0.041 mmol/l VB_2_, a known volume of PFOS solution (*c*_L0_) and deionized water were pipeted into the dialysis bag (Fig. 8). After 10 hours at 37°C, 2.5 ml of the dialysis solution was collected from the sampling tube and the concentration of VB_2 _was determined. The binding ratio of VB_2 _to SA was calculated to investigate the effect of PFOS on the transport function of SA.

## Authors' contributions

XZ performed research and analyzed data; LC provided the detection method; XCF and YSM assisted to write and revise the paper and HWG designed research and wrote the paper. All authors read and approved the final manuscript.

## Supplementary Material

Additional file 1 **Determination results of PFOS by the CPC-ECR colorimetric method and HPLC-MS (n = 3)**. The CPC-ECR colorimetric method for PFOS determination was validated by using HPLC-MS. Click here for file

Additional file 2 **Determination of PFOS by HPLC-MS**. The retention time (A) and the standard curve (B) of PFOS determined by HPLC-MS. Click here for file
