# Supplementary material for: Binding of PFOS to serum albumin and DNA: insight into the molecular toxicity of perfluorochemicals
Source: BMC Mol Biol. 2009 Feb 25;10:16. doi: 10.1186/1471-2199-10-16 (PMC2656506; doi:10.1186/1471-2199-10-16)
Supplement: Additional file 1 — Determination results of PFOS by the CPC-ECR colorimetric method and HPLC-MS (n = 3). The CPC-ECR colorimetric method for PFOS determination was validated by using HPLC-MS. [file 1471-2199-10-16-S1.pdf]

Table 1

Determination Results of PFOS by the CPC-ECR colorimetric method and HPLC-MS (n=3)

$C_{L0,\ PFOS}$ (mg/l)	CPC-ECR (mg/l)	HPLC-MS (μg/l)
100.0	18.30±0.121	17.77±0.003
150.0	59.42±0.022	57.77±0.003
200.1	88.79±0.034	87.50±0.004
