# Supplementary material for: Binding of PFOS to serum albumin and DNA: insight into the molecular toxicity of perfluorochemicals
Source: BMC Mol Biol. 2009 Feb 25;10:16. doi: 10.1186/1471-2199-10-16 (PMC2656506; doi:10.1186/1471-2199-10-16)
Supplement: Additional file 2 — Determination of PFOS by HPLC-MS. The retention time (A) and the standard curve (B) of PFOS determined by HPLC-MS. [file 1471-2199-10-16-S2.pdf]

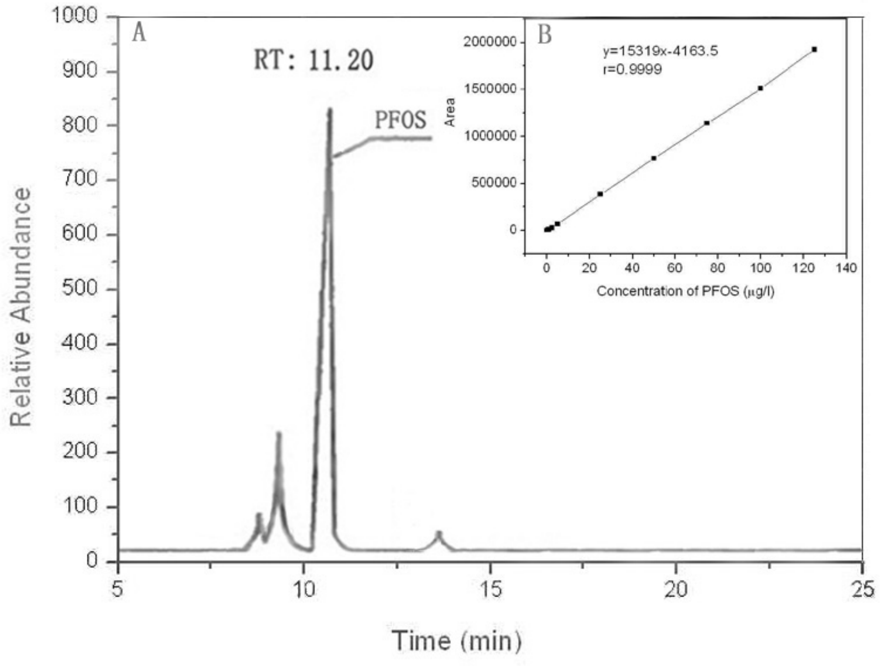

Additional Figure Determination of PFOS by HPLC-MS.

Referrence

Kannan, K; Choi, J; Iseki, N; Senthilkumar, K; Kim, DH; Masunaga, S; Giesy, JP. **Concentrations of perfluorinated acids in livers of birds from Japan and Korea.** *Chemosphere* 2002c, **49:**225-231.
